# Atypical Takotsubo Cardiomyopathy Precipitated by Gastrointestinal Bleeding: Review of the Pathophysiology

**DOI:** 10.7759/cureus.34515

**Published:** 2023-02-01

**Authors:** Aniekeme S Etuk, Celestine I Odigwe, Christabel Nyange, Stanley Thornton, Michael Pursley

**Affiliations:** 1 Internal Medicine, Thomas Hospital - Infirmary Health, Fairhope, USA; 2 Cardiovascular Disease, Morehouse School of Medicine, Atlanta, USA; 3 Cardiovascular Disease, Thomas Hospital - Infirmary Health, Fairhope, USA

**Keywords:** stress induced cardiomyopathy, left ventricular dysfunction, gastrointestinal bleed, cardiomyopathy, atypical takotsubo

## Abstract

Stress-induced cardiomyopathy, otherwise known as takotsubo cardiomyopathy, typically presents with chest pain and acute left ventricular failure with unobstructed coronary arteries. There is an increase in disease incidence as clinicians are becoming more aware of this clinical entity. An atypical variant exists where there is left ventricular dysfunction with apical sparing. Various precipitants have been described in the literature, however, there has not been any documented case following massive gastrointestinal bleeding. We report an atypical variant of takotsubo cardiomyopathy following a gastrointestinal bleed with review of the pathophysiologic mechanisms behind the disease process.

## Introduction

Takotsubo cardiomyopathy is described as a transient dyskinesia, hypokinesia or akinesia of the left ventricle with apical ballooning in the absence of obstructive coronary artery disease with electrocardiogram changes and elevation of cardiac enzymes [1]. It was first described in Japan in 1990 where it got its name “Tako-Tsubo” meaning “octopus trap” [2]. Although its incidence is on the rise, it still poses a diagnostic dilemma as it presents as a great mimicker [3]. Variants with apical sparing and mid-ventricular akinesis have also been described, thus referred to as an atypical form [3]. The pathophysiology is unclear, however, a variety of triggers have been implicated [4]. We present a case of atypical takotsubo cardiomyopathy precipitated by gastrointestinal hemorrhage.

## Case presentation

**Figure 1:**
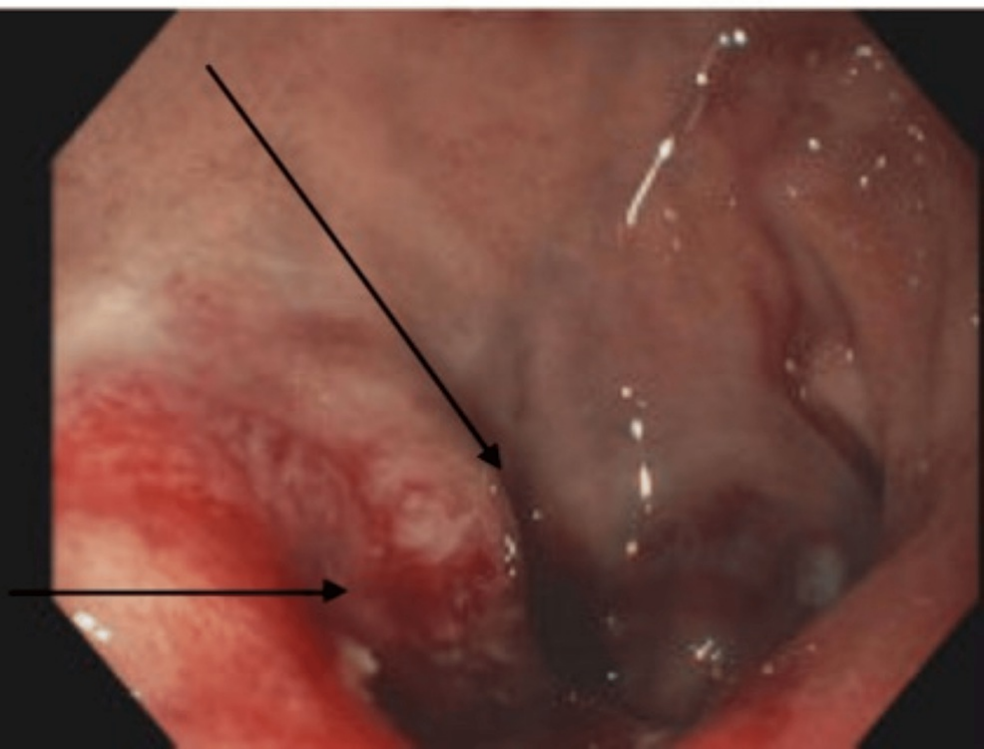
Picture of colonoscopy showing bleeding mucosal ulcer in the sigmoid colon (see black arrows)

A 73-year-old female developed chest pain, palpitations, and shortness of breath of one-day duration. She was hospitalized 48 hours prior for bloody diarrhea, associated with nausea, vomiting, and near syncopal episode. The patient received two units of packed red blood cells during that admission and had a colonoscopy revealing a bleeding ulcerated mucosa in the sigmoid colon (Figure 1). She had a past medical history of primary hypertension, hypothyroidism, and osteoarthritis. Additional history was also significant for the loss of spouse over a year ago. On physical examination, the patient was tachycardic with a heart rate of 118 beats per minute. Heart sounds were normal with no murmurs.

**Figure 2:**
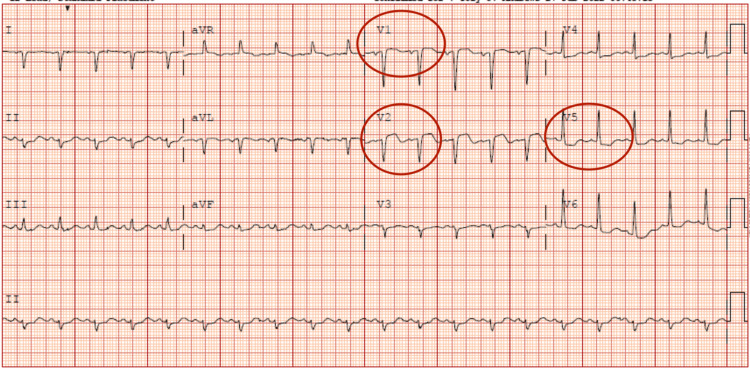
EKG showing ST elevation in V1, and V2 precordial leads with ST depression in V5 (see red circles).

**Figure 3:**
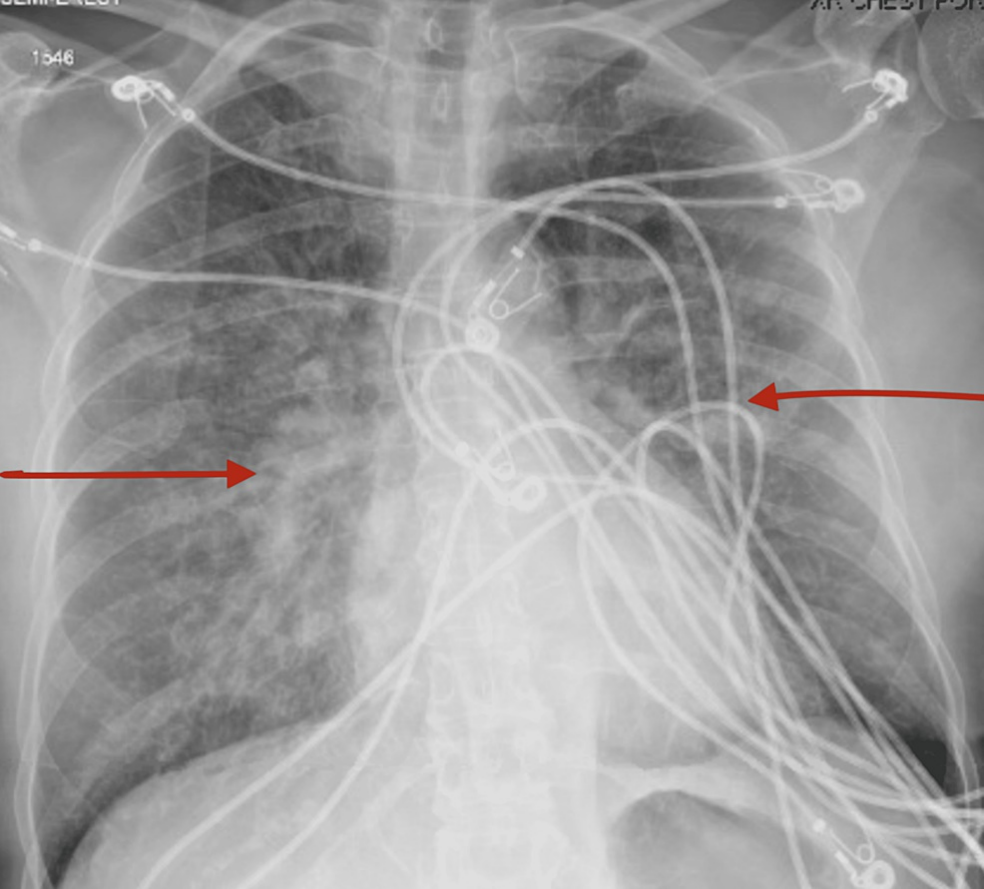
Chest X-ray showing bilateral pulmonary infiltrates (see red arrows).

Complete metabolic panel and complete blood count were carried out and a relevant comparison with previous results was done (Table 1). Electrocardiogram on presentation showed ST elevation in the precordial leads (Figure 2) and troponin T was elevated at 0.08 ng/mL (0.00 - 0.01). She was also noted to have an elevated proBNP of 37,000 pg/mL (0 - 125). Chest X-ray showed bilateral pulmonary infiltrates (Figure 3). The patient subsequently underwent coronary angiography with findings of a moderate non-obstructive coronary artery stenosis (Videos 1, 2) with an ejection fraction of 25-30%. An echocardiogram done to elucidate the etiology of her cardiomyopathy revealed severe hypokinesis of the mid-left ventricular segments, hypokinesia of the base and apex of the left ventricle with an ejection fraction of 40% (Video 3). Previous echocardiogram done six months prior had shown a normal left ventricular systolic function with an ejection fraction of 60-65% and an absence of wall motion abnormalities.

**Table 1:** Comparison table of some relevant complete metabolic panel and complete blood count parameters

Parameters	Prior to gastrointestinal bleeding	At presentation with gastrointestinal bleeding	At presentation with atypical takotsubo cardiomyopathy
White blood cell (K/ul)	10.8 (4.5 – 12.0)	7.2 (4.5 – 12.0)	9.5 (4.5 – 12.0)
Hemoglobin (g/dL)	14.0 (11.5 – 16.0)	6.8 (11.5 – 16.0)	10.2 (11.5 – 16.0)
Hematocrit (%)	41.8 (35.0 – 48.0)	20.4 (35.0 – 48.0)	32.9 (35.0 – 48.0)
Platelet count (K/ul)	290 (120 – 450)	178 (120 – 450)	182 (120 – 450)
Sodium (mmol/L)	139 (135 – 145)	137 (135 – 145)	140 (135 – 145)
Potassium (mmol/L)	4.2 (3.5 – 5.1)	4.3 (3.5 – 5.1)	4.1 (3.5 – 5.1)
Creatinine (mg/dL)	1.07 (0.51 – 0.95)	2.20 (0.51 – 0.95)	1.20 (0.51 – 0.95)

**Video 1:** Left coronary angiography showing moderate non-obstructive coronary artery stenosis.

**Video 2:** Right coronary angiography showing moderate non-obstructive coronary artery stenosis.

**Video 3:** Echocardiogram showing severe hypokinesis of all the mid-left ventricular segments, hypokinesia of the base and apex of the left ventricle with an ejection fraction of 40% (see red arrow).

Based on the symptoms at presentation, electrocardiogram findings and elevated troponin, ST segment elevation myocardial infarction was suspected. Chest X-ray findings were consistent with pulmonary edema. A diagnosis supported by elevated proBNP and clinical presentation. An assessment of acute heart failure secondary to probable acute coronary syndrome was made. Her negative coronary angiogram raised concerns for other causes of acute heart failure. Transthoracic echocardiogram confirmed atypical takotsubo cardiomyopathy. Gastrointestinal hemorrhage was the historical precipitating factor. Also, computed tomography angiography of the chest with intravenous contrast was negative for pulmonary embolic disease and aortic dissection.

The patient was started on dopamine infusion together with intravenous furosemide, for cardiogenic shock which developed few hours following hospitalization. She was monitored closely in the medical intensive care unit and eventually weaned off pressor support. Goal-directed medical therapy for heart failure with losartan 25 mg daily and metoprolol 25 mg daily was also instituted.

She improved clinically and was subsequently discharged home. She was seen in the outpatient service a week after discharge and was found to be in a stable clinical condition. Follow-up appointment scheduled in three months.

## Discussion

Takotsubo cardiomyopathy, also termed as broken heart syndrome, ampulla cardiomyopathy or stress-induced cardiomyopathy have all been used to describe left ventricular systolic dysfunction presenting with apical akinesis and ballooning. Although apical akinesis is classic in takotsubo cardiomyopathy, other subtypes with mid-left ventricular, basal akinesia or hypokinesia with apical sparing have also been described [3]. The Mayo clinic revised criteria are used in the diagnosis of takotsubo cardiomyopathy. Criteria include the absence of coronary artery disease on angiography, transient dyskinesis, hypokinesis or akinesis of the left ventricle mid-segment with or without apical involvement, electrocardiogram evidence of ST segment elevation and/or T wave inversion, modest elevation of troponin and absence of myocarditis or pheochromocytoma [1].

The precise pathophysiology is unclear. Some postulations involving plasma catecholamine and neuropeptides stimulating beta 2 coupling from Gs to G1, leading to negative inotropy and left ventricular dysfunction have been made [1,4]. This phenomenon is called stimulus trafficking and explains the typical variant of takotsubo cardiomyopathy as beta-adrenergic receptors are predominant in the apical region of the left ventricle [1,4]. Estrogen deficiency has been described as another pathway in the development of takotsubo cardiomyopathy as 90% of cases occur in postmenopausal women [1,4]. The catecholamine theory in the development of takotsubo cardiomyopathy is most widely recognized and is typically triggered by a stressful event, emotional or physical [1].

Various triggers have been reported including ischemic stroke [3], amiodarone-induced hyperthyroidism [5], and post-surgical procedures. Our patient presented with chest pain and electrocardiographic findings concerning for ST segment myocardial infarction. This was ruled out by the normal subsequent coronary angiogram. Further work-up revealed echocardiographic findings of mid-ventricular akinesis, hypokinesis of the base with apical sparing in keeping with an atypical variant of takotsubo cardiomyopathy. She had a two-day preceding hospitalization for acute blood loss anemia and presyncope requiring transfusion. A colonoscopy done afterward showed ulceration to the mucosa of the sigmoid colon. This will serve as the first reported case of atypical takotsubo cardiomyopathy triggered by gastrointestinal bleeding. The index patient presenting with gastrointestinal bleed, with near syncope and no clinical evidence of shock at the initial hospitalization, makes cardiogenic shock less likely the culprit of the mucosal ulcer noted on colonoscopy.

The exact mechanism for the development of the atypical variant of takotsubo cardiomyopathy following gastrointestinal bleeding is unclear, however, we believe that the catecholamine surge following massive blood loss, resulted in upregulation of the beta-adrenergic receptors, causing the regional myocardial dysfunction. Our patient was also post-menopausal, thus losing the cardio-protective effect that estrogen confers, further predisposing her to the condition.

## Conclusions

Takotsubo cardiomyopathy still poses a challenge with regard to its diagnosis. It should be considered in the differential in those who present with what seemingly looks like a myocardial infarction. Echocardiogram plays a vital role in its diagnosis as it aids in highlighting characteristic wall motion abnormalities. Conditions causing increased catecholamine release have been established to be the main culprit. It is therefore necessary for healthcare providers to be aware of massive gastrointestinal bleeding as a potential precipitating factor.

## References

[1] Khalid N, Ahmad S, Shlofmitz E, Chhabra L (2022). Pathophysiology of takotsubo syndrome. In: StatPearls [Internet].

[2] Dote K, Sato H, Tateishi H, Uchida T, Ishihara M (1991). Myocardial stunning due to simultaneous multivessel coronary spasms: a review of 5 cases [Article in Japanese]. J Cardiol.

[3] Loong CW, Firdaus MA, Said MR, Abidin IZ (2020). Atypical presentation of takotsubo cardiomyopathy: stroke as a predisposing factor. Medeni Med J.

[4] Lyon AR, Citro R, Schneider B, Morel O, Ghadri JR, Templin C, Omerovic E (2021). Pathophysiology of takotsubo syndrome: JACC state-of-the-art review. J Am Coll Cardiol.

[5] Capel I, Tasa-Vinyals E, Cano-Palomares A, Bergés-Raso I, Albert L, Rigla M, Caixàs A (2017). Takotsubo cardiomyopathy in amiodarone-induced hyperthyroidism. Endocrinol Diabetes Metab Case Rep.

